# Supplementary material for: Bright and stable anti-counterfeiting devices with independent stochastic processes covering multiple length scales
Source: Nat Commun. 2025 Jan 8;16:502. doi: 10.1038/s41467-024-55646-4 (PMC11711641; doi:10.1038/s41467-024-55646-4)
Supplement: Supplementary file 1 — Supplementary Information [file 41467_2024_55646_MOESM1_ESM.pdf]

Supporting Information

Bright and stable anti-counterfeiting devices with independent stochastic processes covering multiple length scales

Junfang Zhang, Adam Creamer, Kai Xie, Jiaqing Tang, Luke Salter, Jonathan P. Wojciechowski, Molly M. Stevens

Email: molly.stevens@dpag.ox.ac.uk

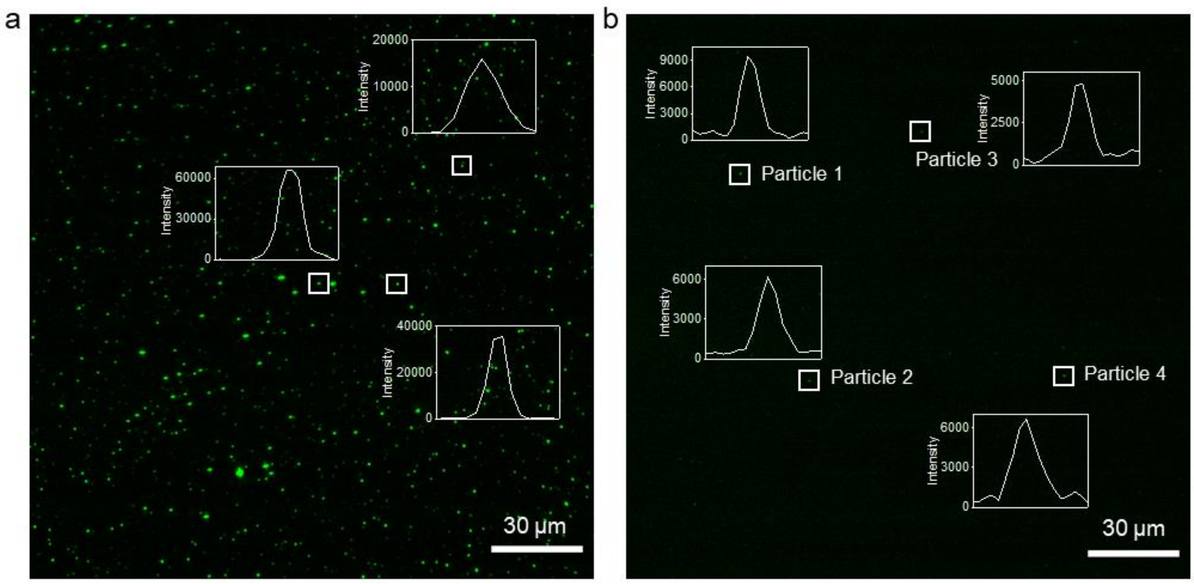

Supplementary Figure 1. Fluorescent images of dried nanoparticles. Images of a) F8BT NPs and b) Qdots 525 (laser power:100%, gain: 50). Both F8BT NPs and Qdots (CdSe:ZnS) were excited at 405 nm. F8BT NPs were prepared with the condition of 50 μL PS-g-FEO and 1000 μL milli-Q water. The curves show the fluorescence intensity of the particles in the frames.

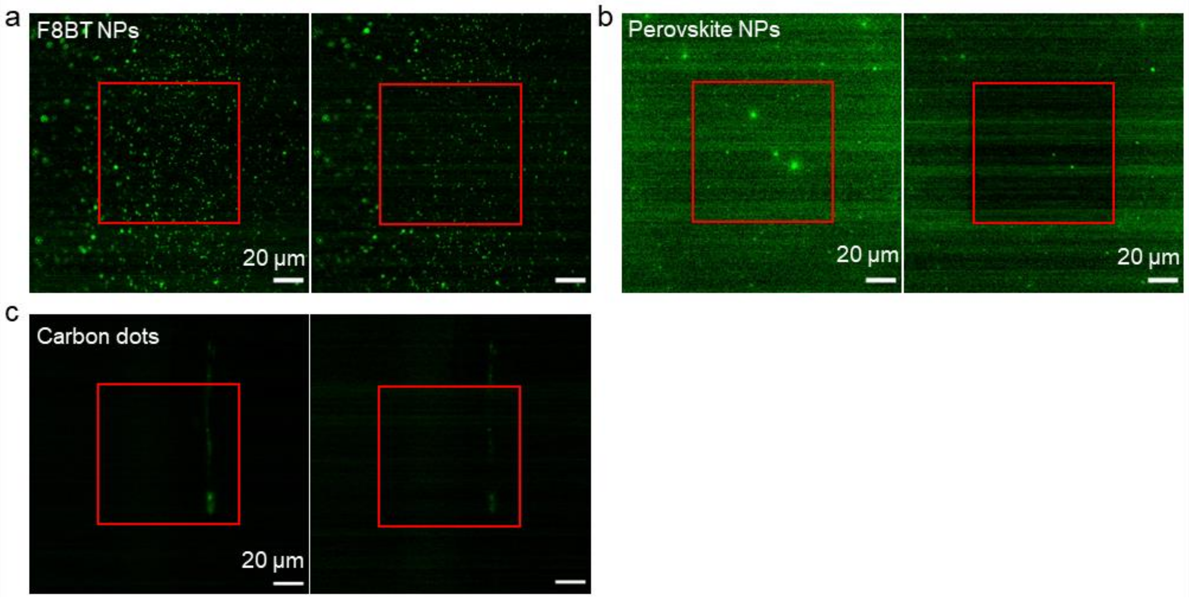

Supplementary Figure 2. Photobleaching test of dried nanoparticles. Confocal images of a) F8BT NPs (Ex: 405 nm, laser power: 15%, gain: 150), b) perovskite ($CsPbBr_3$) NPs (Ex: 405 nm, laser power: 15%, gain: 150), and c) carbon dots (Ex: 405 nm, laser power: 15%, gain: 500) before and after photobleaching. The whole images were considered, and all fluorescence signal was extracted. Detailed algorithms for picture processing can be found in Supplemental Materials.

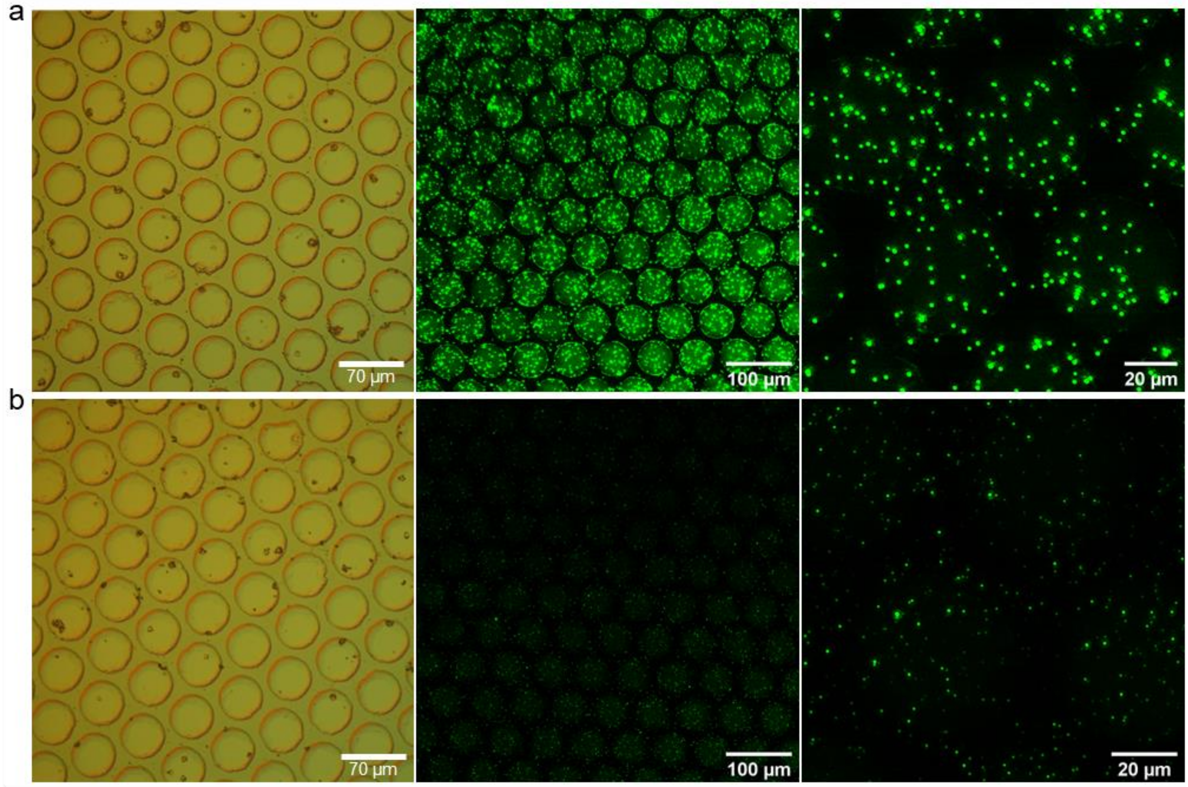

Supplementary Figure 3. Optical and confocal images of the printed microspots embedded with F8BT NPs. The synthesis condition in a) is F8BT: S=5;1, 0.25 mL H_2O, diluted by the same volume of ethanol. And the condition in b) is F8BT: S=5;5, 1 mL H_2O, no dilution. Laser power: 10%, gain: 100%.

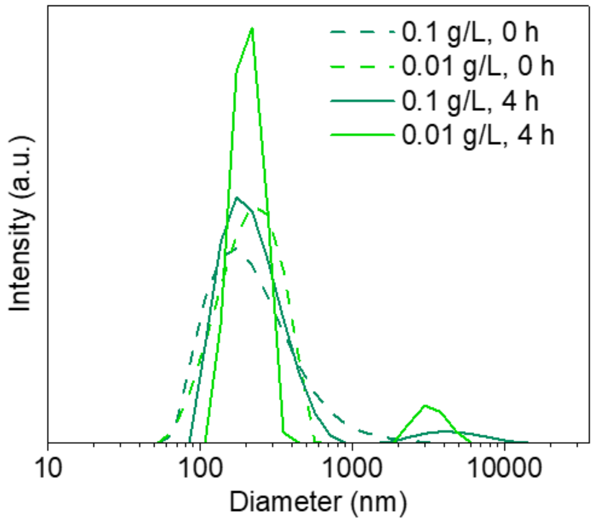

Supplementary Figure 4. DLS intensity distribution of F8BT nanoparticles. Solutions with different concentrations (F8BT: Surfactant = 5:1) before and after 4 h.

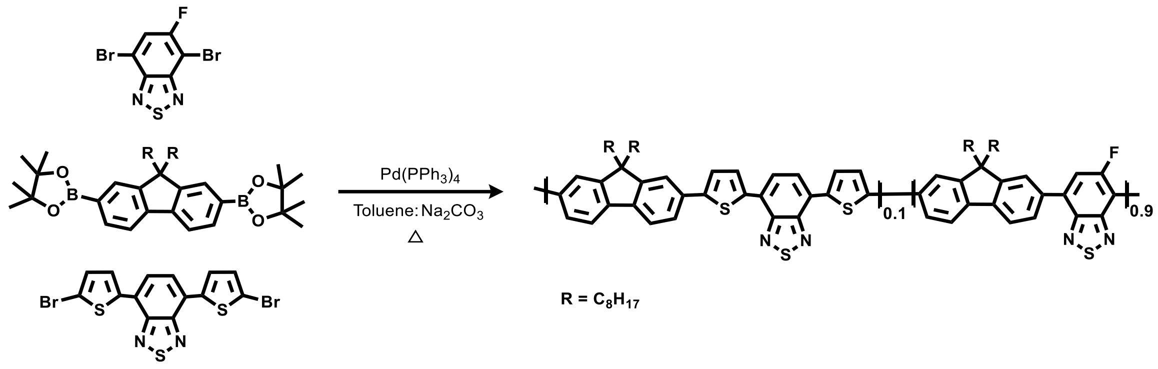

Supplementary Figure 5a. Scheme of F8BT-red synthesis.

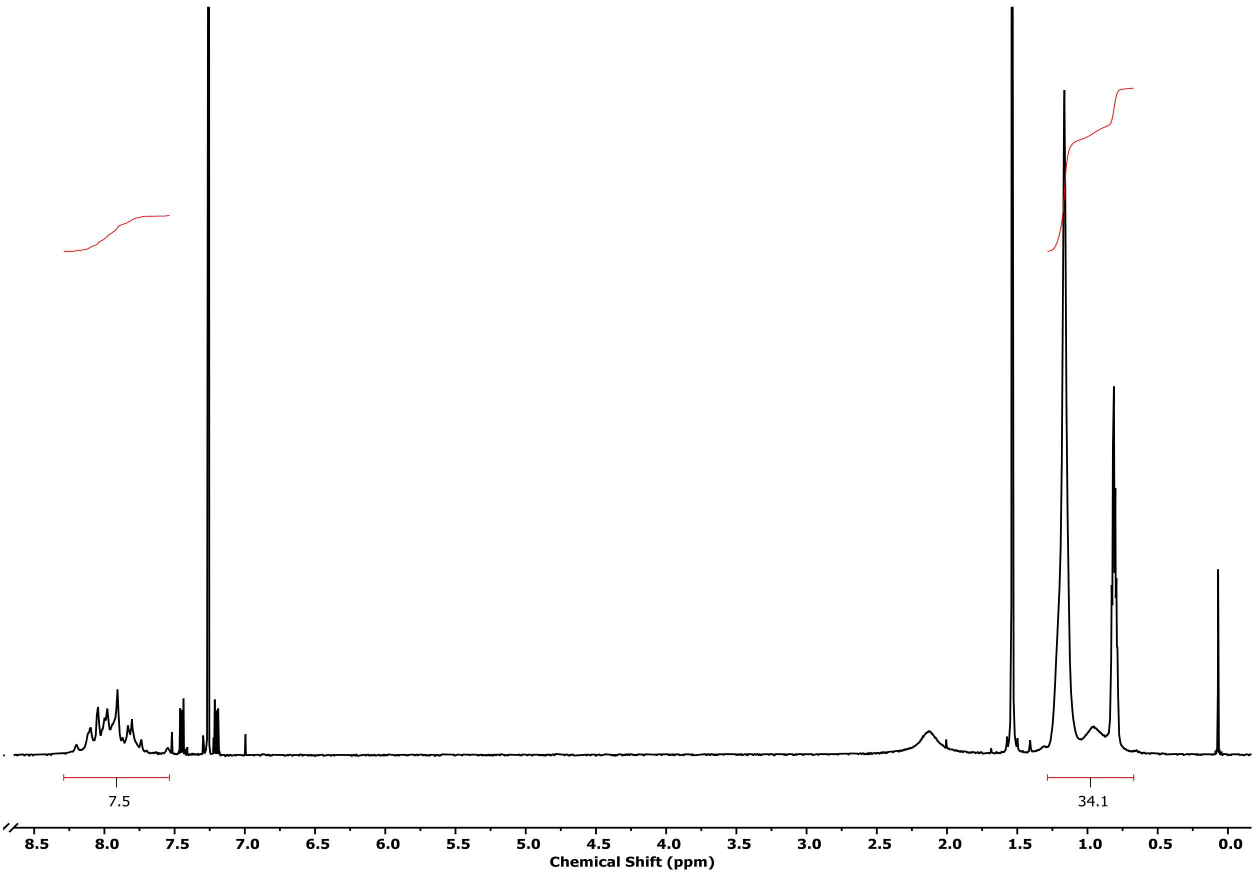

Supplementary Figure 5b. **^{1}H NMR spectra of F8BT-red in $CDCl_3$ with interpreted integration values.**

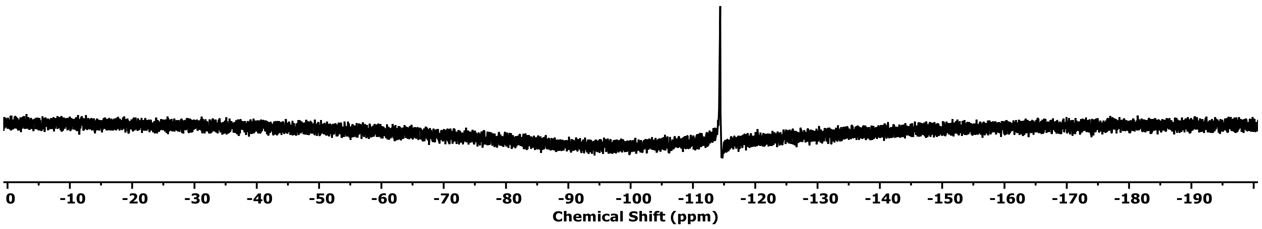

Supplementary Figure 5c. **^{19}F NMR spectra of F8BT-red in $CDCl_3$**

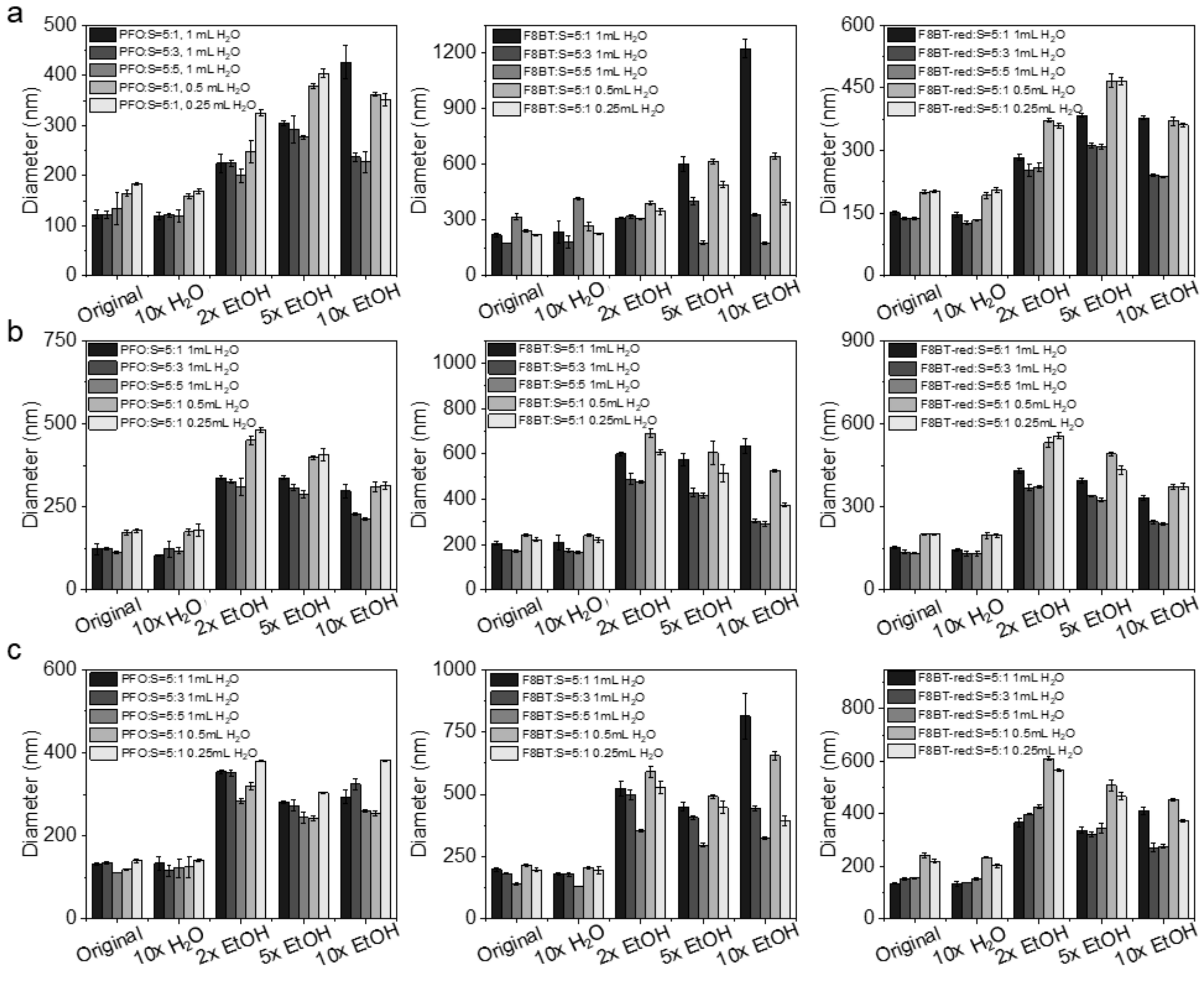

Supplementary Figure 6. Nanoparticle diameter extracted from DLS. The synthesis process and size characterization of SPNs have been independently repeated three times. DLS analysis of the nanoparticles from a) the first synthesis, b) the second synthesis, and c) the third synthesis.

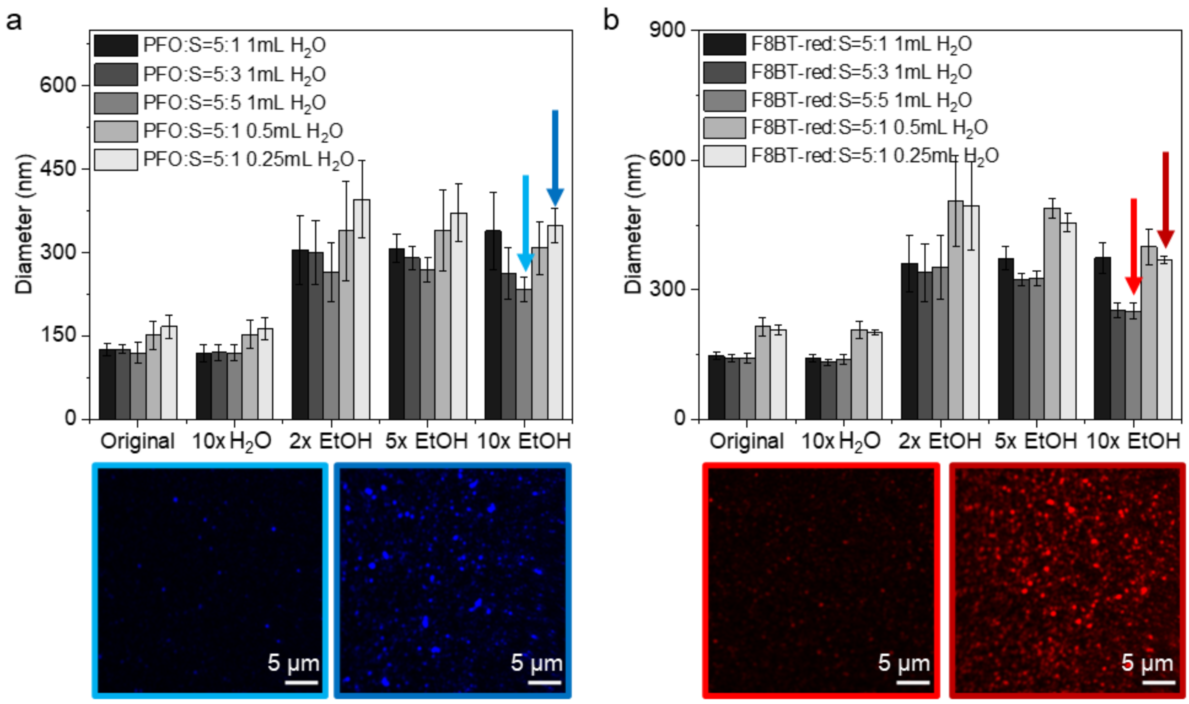

Supplementary Figure 7. DLS analysis of blue and red nanopartilces. a) PFO and b) F8BT-red nanoparticles (N=3, S: surfactant poly(styrene)-graft-poly(ethylene oxide)). For PFO nanoparticles, the confocal images were obtained under 20% laser power and a gain value of 100 (Ex 405 nm. Em 450-600 nm). For F8BT-red nanoparticles, the confocal images were obtained under 10% laser power and a gain value of 50 (Ex 430 nm. Em 550-700 nm).

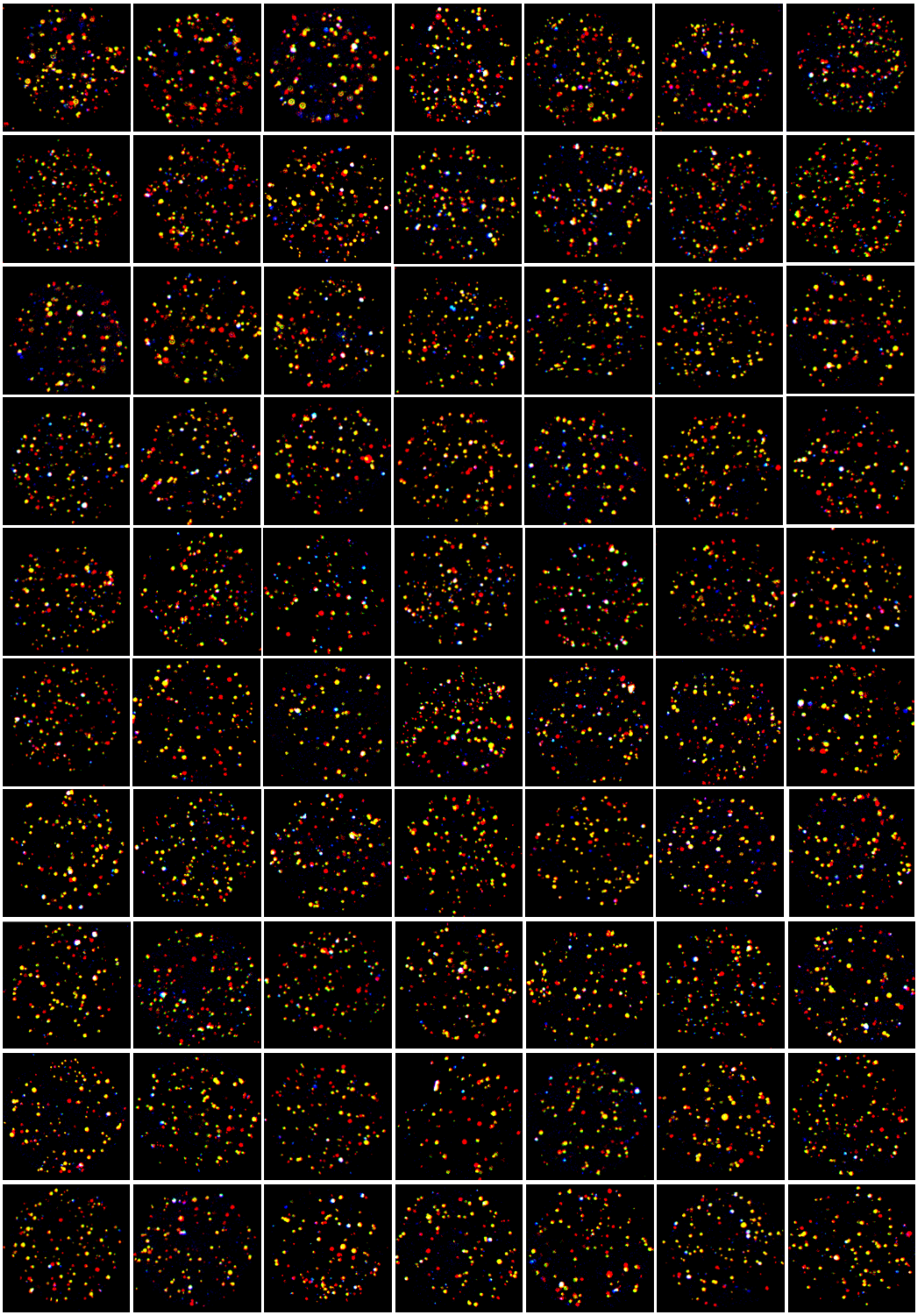

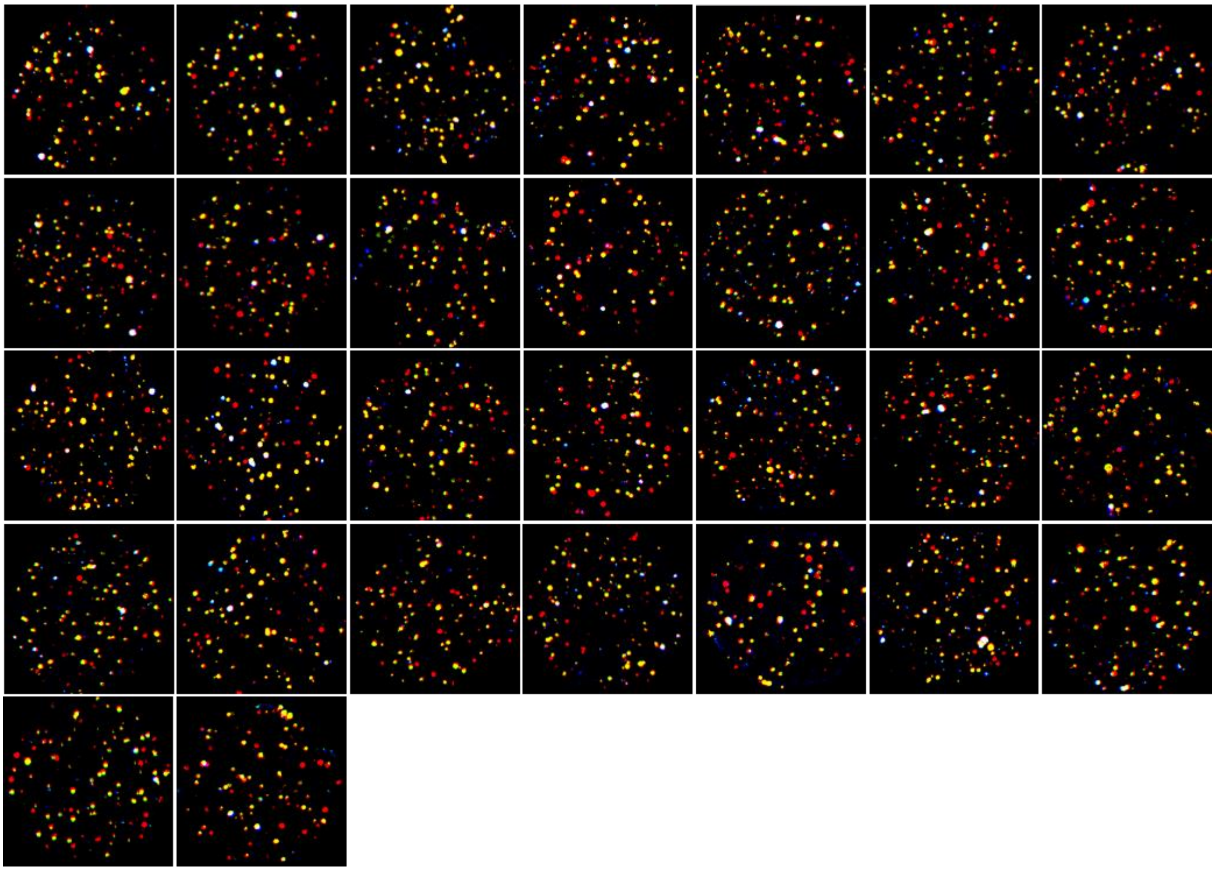

Supplementary Figure 8. Microspots embedded with SPNs (n=100) were scanned by a confocal microspore for statistical analysis. (Blue channel: E_x 405 nm, E_m 430-470 nm, 30% laser power, 150% gain. Green channel: E_x 405 nm, E_m 470-550 nm, 20% laser power, 100% gain. Red channel: E_x 405 nm, E_m 570-700 nm, 20% laser power, 100% gain.)

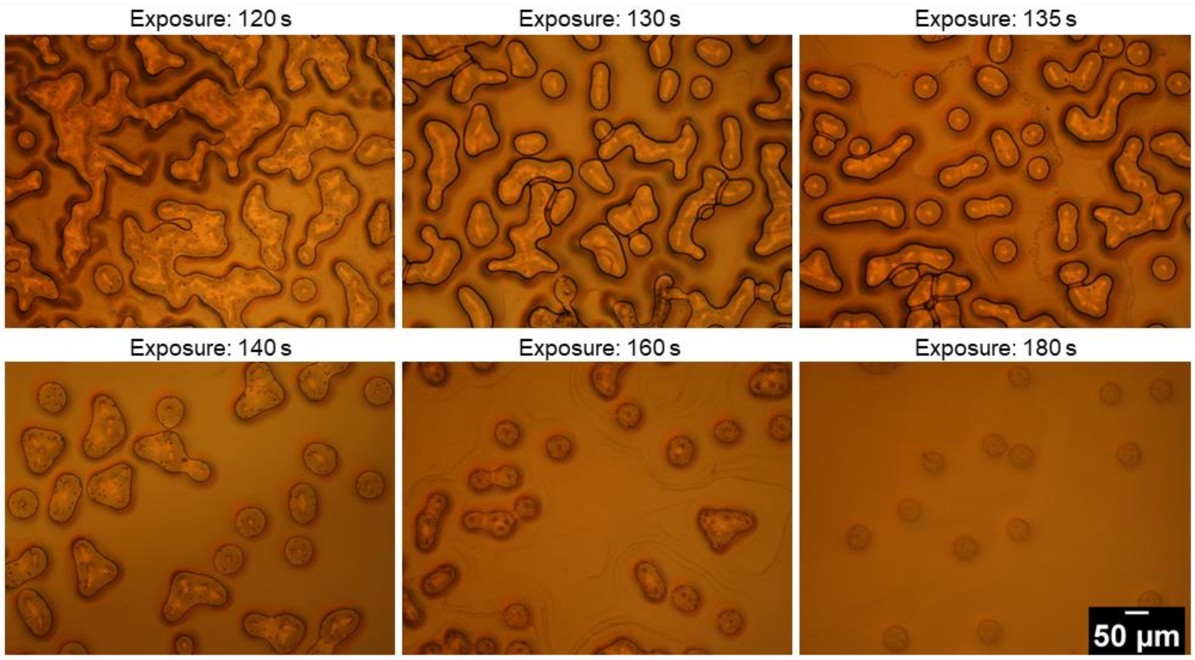

Supplementary Figure 9. Morphologies of AZ 4562 and SU 8 2010 photoresist mixture (1:1.5) with different exposure dose. The exposure process was performed by hard contact under 365 nm UV irradiation with an intensity of 3 mW/cm^2.

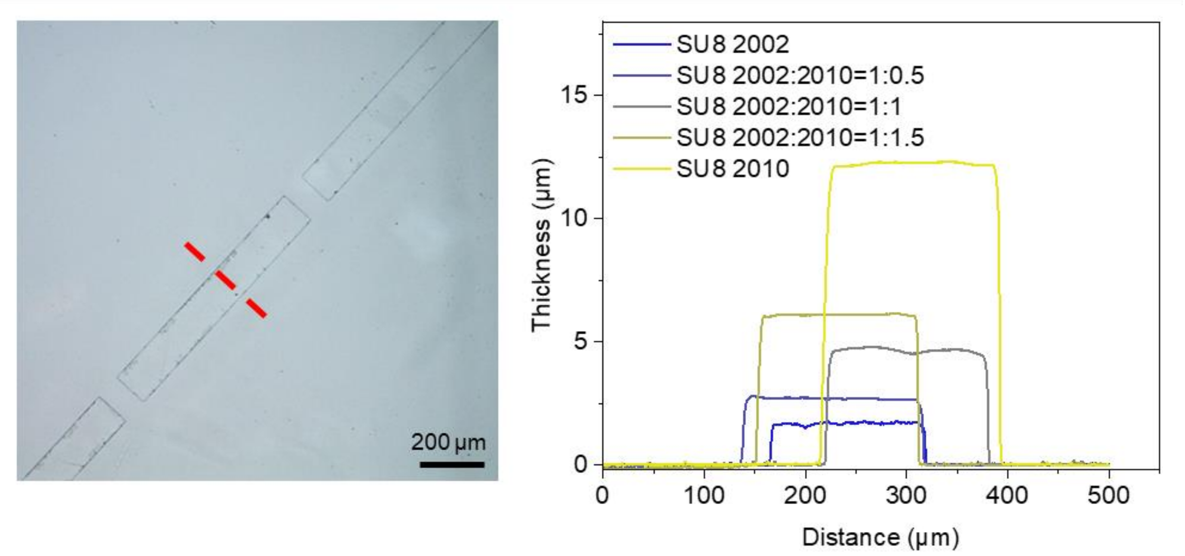

Supplementary Figure 10. Thickness investigation of different photoresist recipes. For all measurements, photoresists were spin-coated onto a glass slide with a speed of 2000 rpm for 30 s. Photolithography process conditions: 1 minute at 75°C for softbake, 200 mJ/cm^2 exposure with a wavelength of 365 nm, 1 minute at 110°C for hardbake, and 5 s development. A profile across the printed rectangular (red dotted line) was generated to show the thickness information.

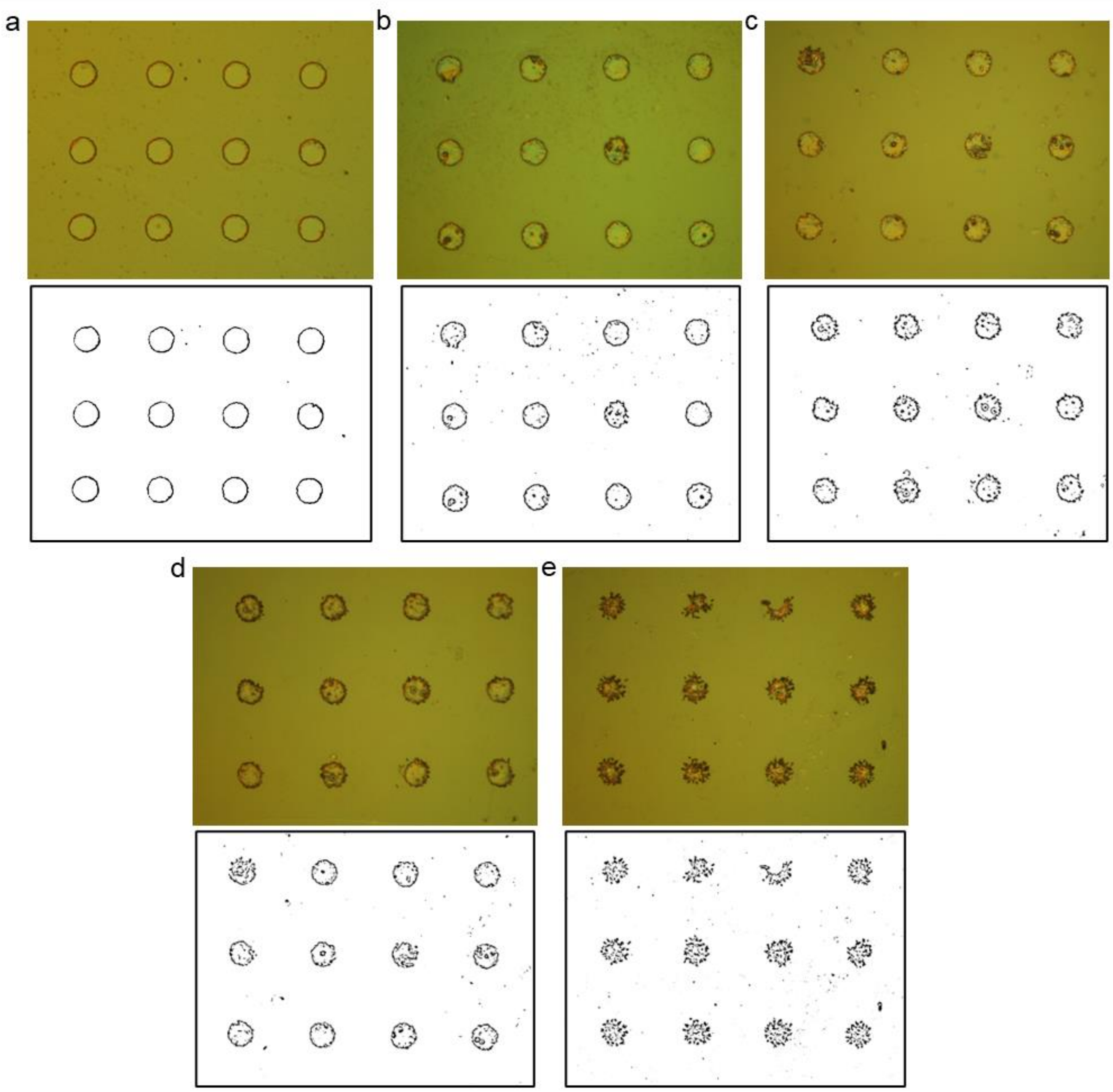

Supplementary Figure 11. Optical images of the printed microspots. Images obtained by a) pure SU 8 2002 and 300 mJ/cm^2 exposure; b) a ratio of 6: 1 between SU 8 2002 and AZ 4562, 300 mJ/cm^2 exposure; c) a ratio of 5: 1 between SU 8 2002 and AZ 4562, 300 mJ/cm^2 exposure; d) a ratio of 4:1 (v/v) between SU 8 2002 and AZ 4562 and 300 mJ/cm^2 exposure; e) a ratio of 4:1 (v/v) between SU 8 2002 and AZ 4562 and 200 mJ/cm^2 exposure, All the exposure was under a wavelength of 365 nm.

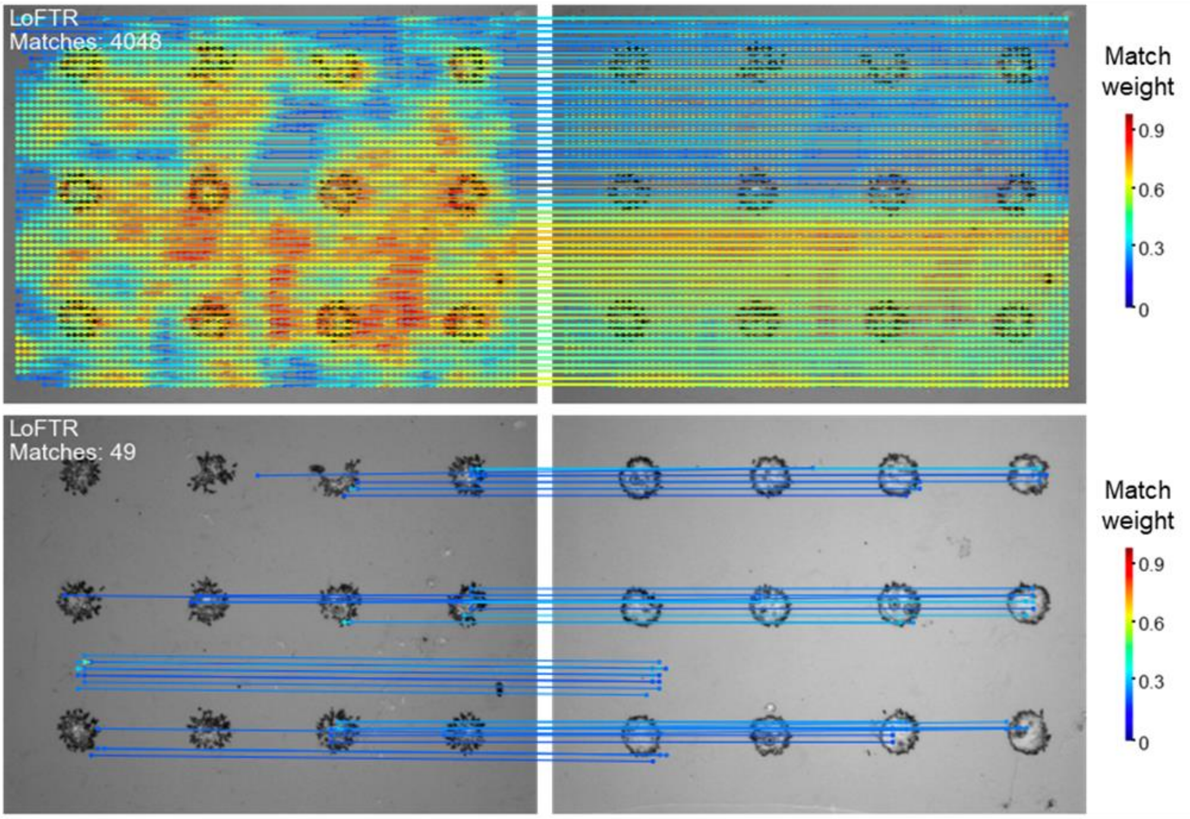

Supplementary Figure 12. Feature matching by the LoFTR algorithm. Analysis of the same (top) and two different (bottom) microspot arrays from two independent scans.

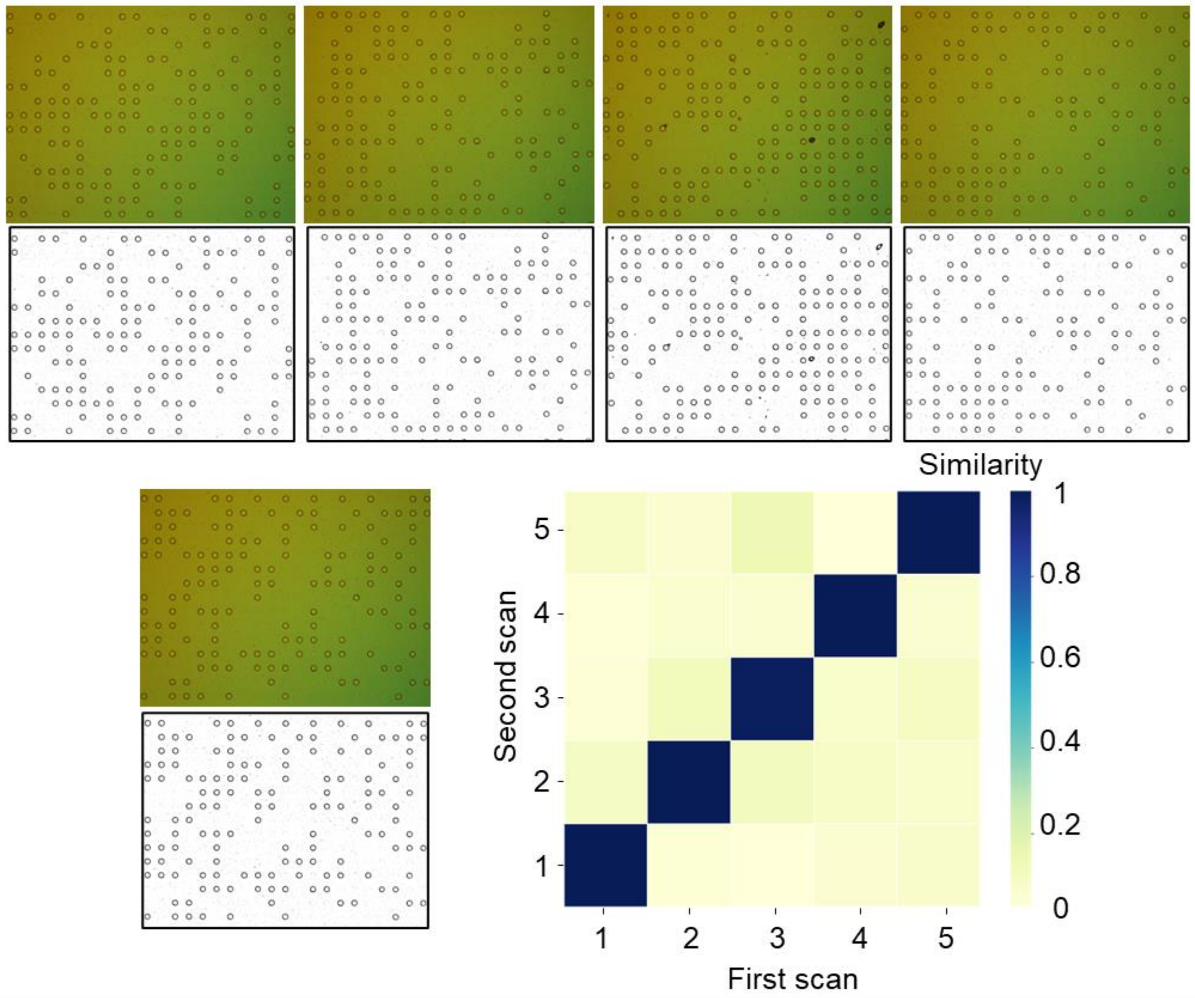

Supplementary Figure 13. Optical pictures of five different arrays with stochastically distributed microspots. By simply assigning the absence or presence of microspots to 0 or 1, the images can be converted to binary codes. The similarities of different scans from the same array and scans of different arrays were calculated.

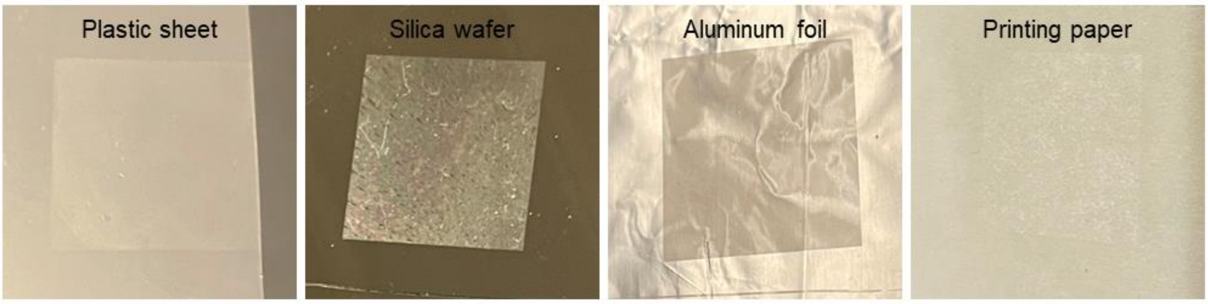

Supplementary Figure 14. Substrate compatibility. The photoresist spot arrays are compatible with different substrates including plastic sheets, silica wafers, aluminum foil, and printing paper.

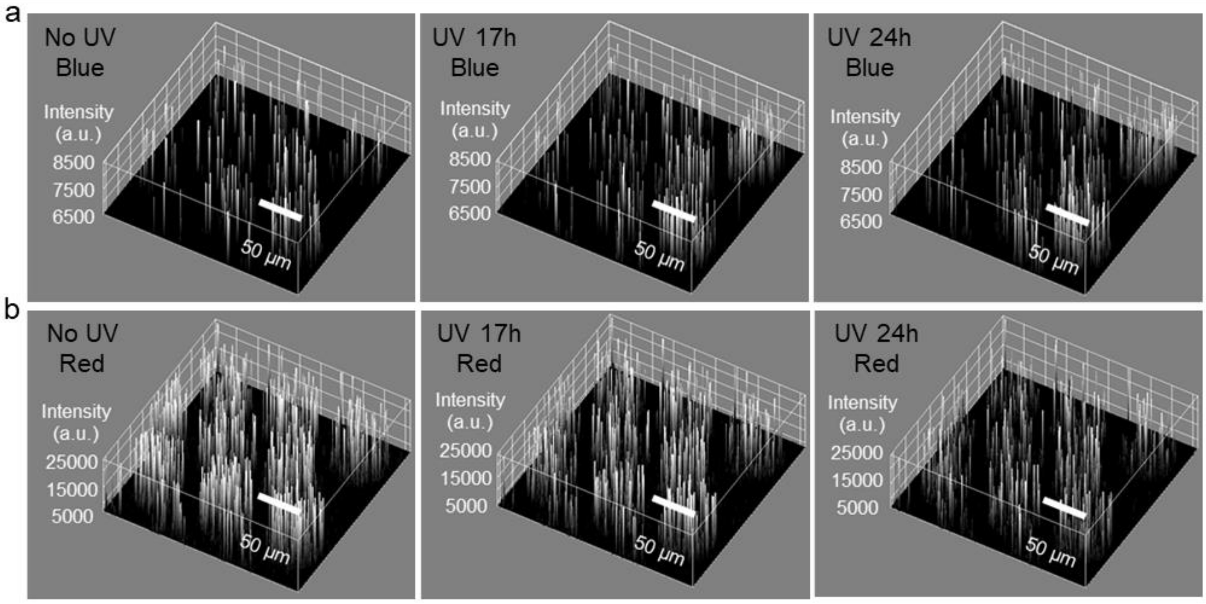

Supplementary Figure 15. Photostability test of the PUF devices. (UV wavelength: 365 nm. UV intensity: 12W.) a) Blue channel: E_x 405 nm, E_m 430-470 nm, 20% laser power, 300% gain. b) Red channel: E_x 405 nm, E_m 650-700 nm, 20% laser power, 300% gain.

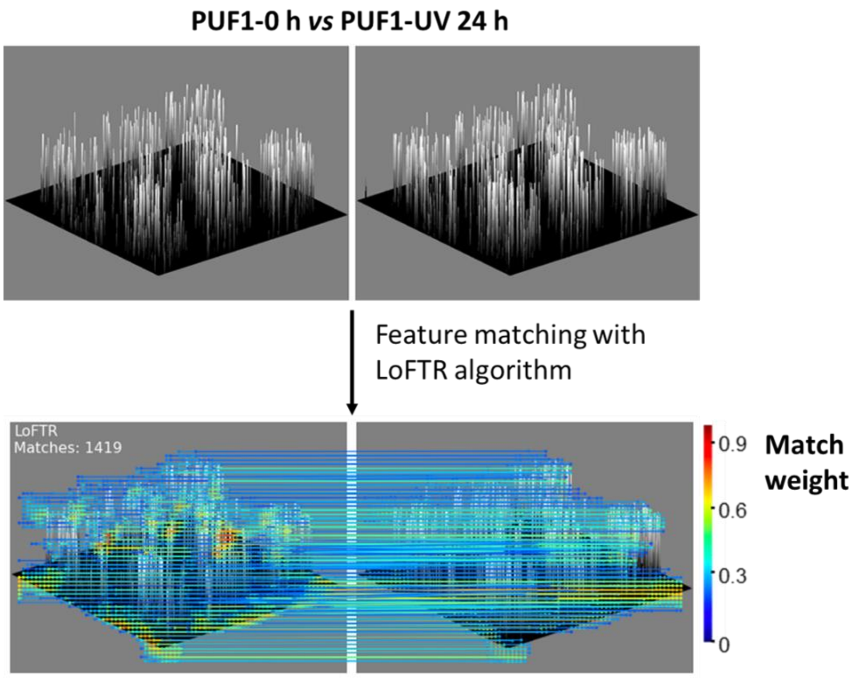

Supplementary Figure 16. An example of feature matching showing the images before and after applying the LoFTR algorithm.

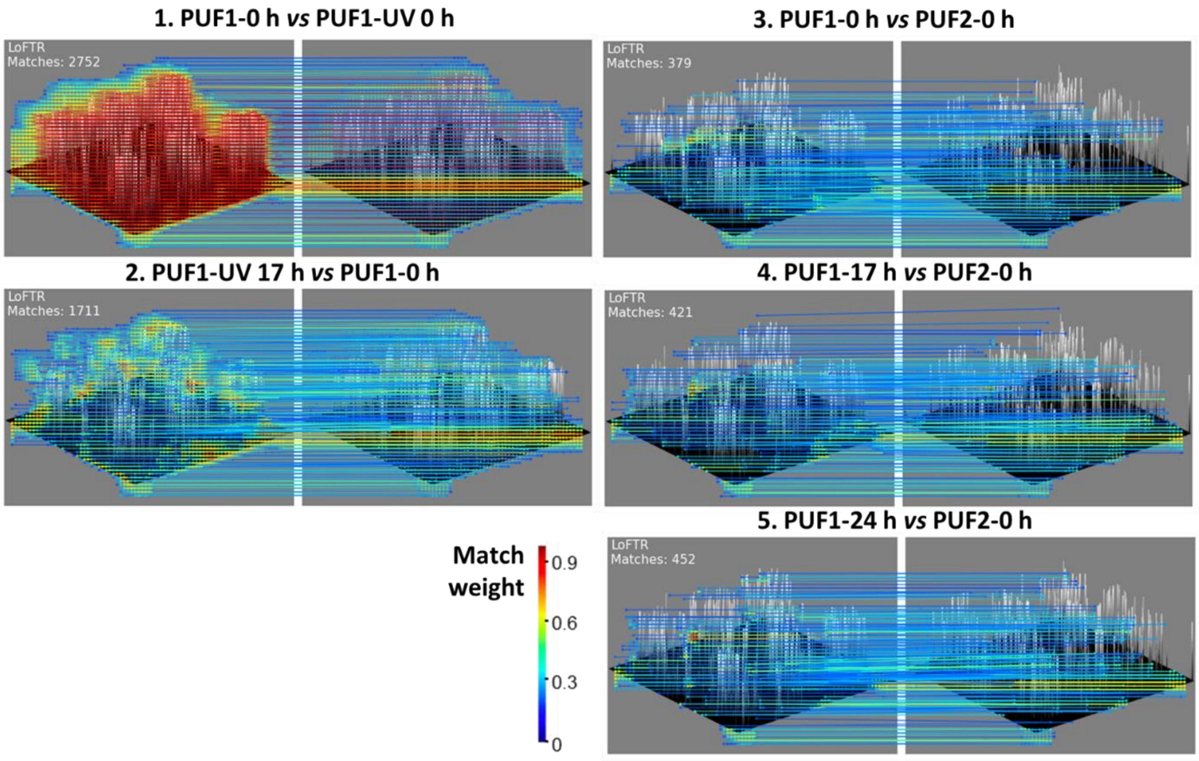

Supplementary Figure 17. Feature matching by the LoFTR algorithm. Analysis of the same (left column) and two different (right column) PUF devices in the green channel.

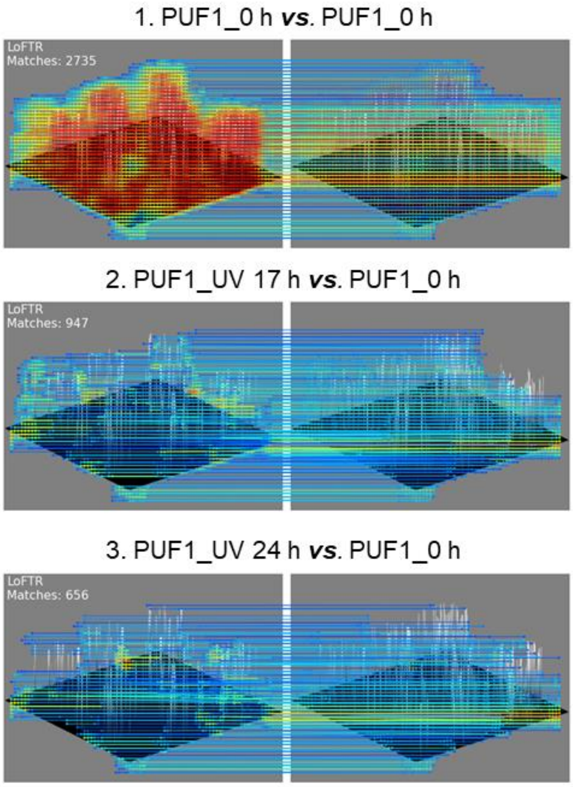

Supplementary Figure 18. Feature matching by the LoFTR algorithm. Analysis of the same (left) and two different (right) PUF devices in the blue channel.

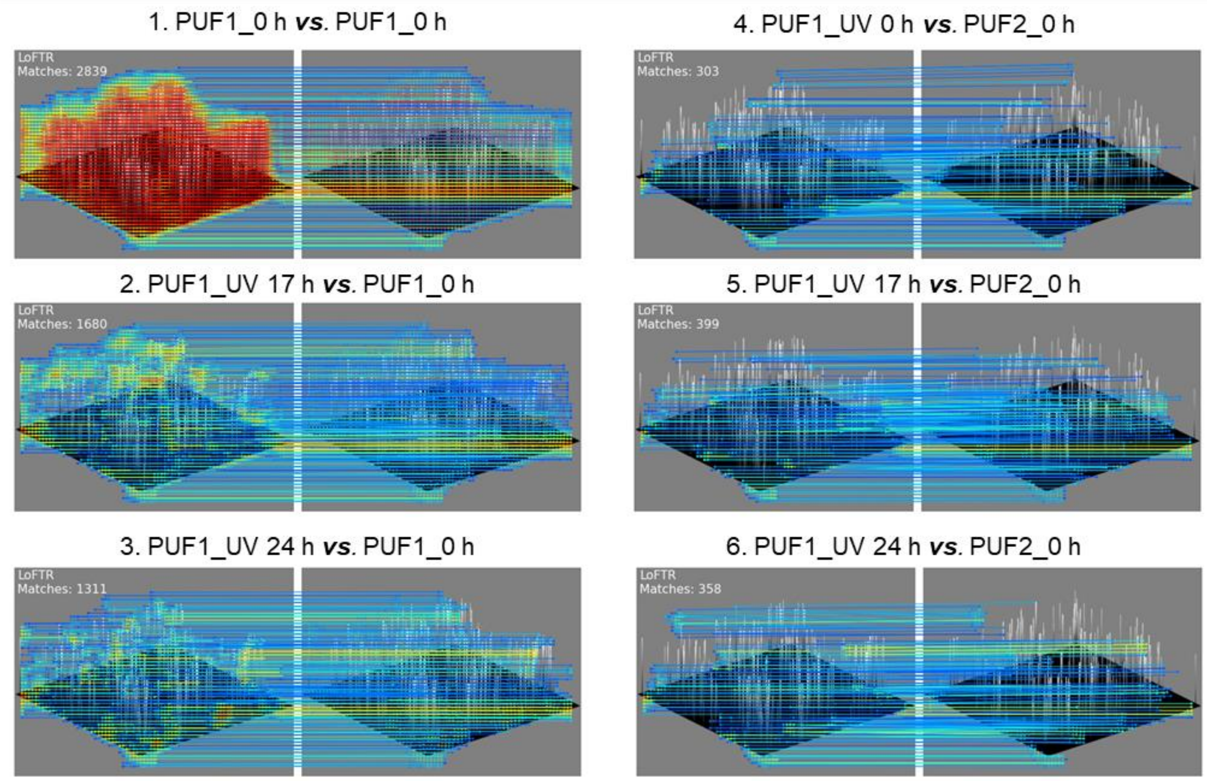

Supplementary Figure 19. **Feature matching by the LoFTR algorithm.** Analysis of the same (left) and two different (right) PUF devices in the red channel.

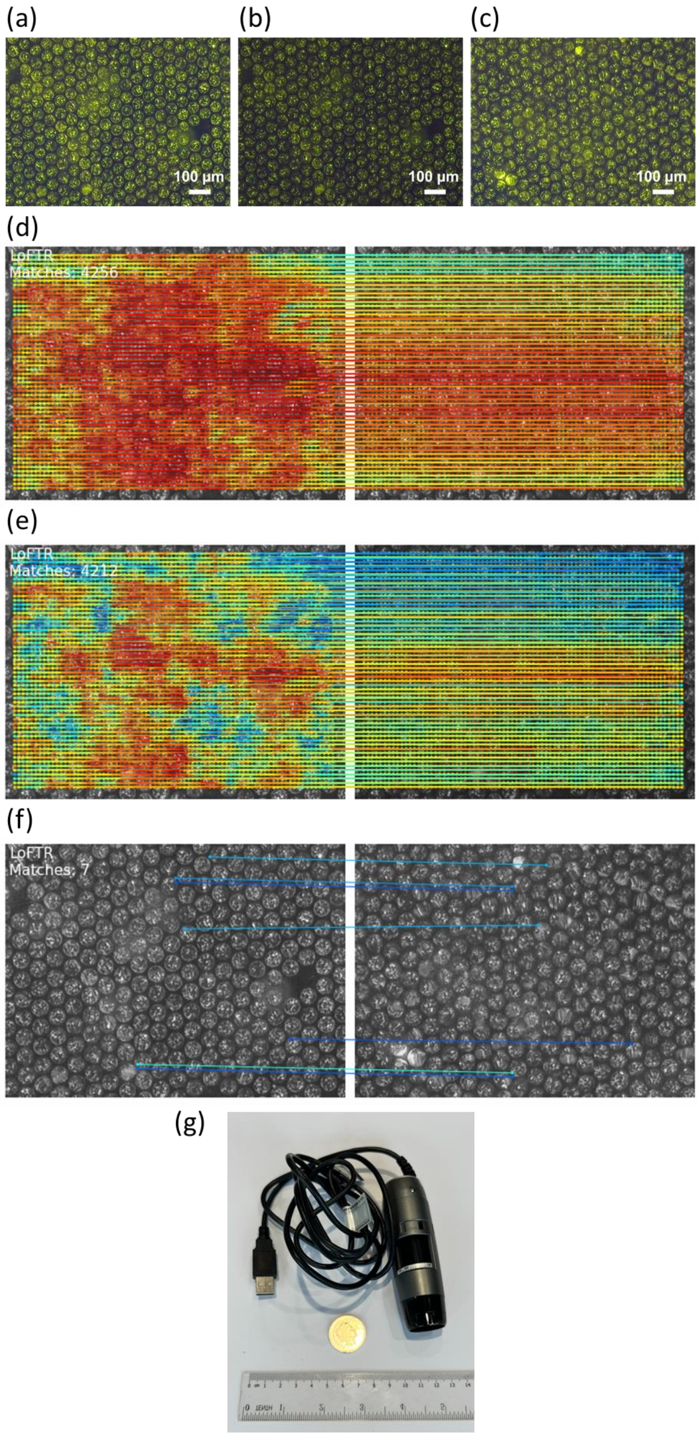

Supplementary Figure 20. Images recorded by a USB microscope and the following feature matching analysis with LoFTR algorithm. Patterns imaged in a) long exposure time (1 s) in area 1, b) short exposure time (1/4 s) also in area 1, c) long exposure time (1 s) in area 2. (d) Feature matching between the two images obtained from same exposure time and area. e) Feature matching between the two images obtained from same PUF but different exposure times. f) Feature matching between the two images obtained from different PUFs recorded with same exposure time. g) Photograph of USB microscope with a ten pence coin for size comparison.

Supplementary Note 1

For calculating the number theoretical challenge-response-pairs (CRPs), the equation is as follows:

$$(1)\ \text{Number of CRPs} = (\#\text{Bits})^{N_{\text{Px}}^{2}}$$

Where N_{Px} is the number of pixels in the image and L_{Px} is the length of each pixel (μm). Therefore, for a 1 x 1 mm image of our three-channel PUF:

$$(2)\ \text{Number of CRPs} = (3+1)^{(1000 \div 0.167)^{2}} \approx 10^{10800129}$$

However, due to sensitivities in small variations in readout, we opted to pack multiple pixels as a unit for digitalization. Specifically, a unit for green and red channels is 10 x 10 pixels, and a unit for blue channel is 50 x 50 pixels. Therefore, the number of CRPs is calculated to be:

$$(3)\ \text{Number of CRPs} = 2^{(1000 \div 0.167 \div 10)^{2}} \times 2^{(1000 \div 0.167 \div 10)^{2}} \times 2^{(1000 \div 0.167 \div 50)^{2}} \approx 10^{220322}$$

Supplementary Note 2

a)

The bit-error-rate (BER) was calculated by the following equation:

$$(4)\quad \mathrm{BER} = \frac{1}{k}\sum_{i=1}^{k}\frac{1}{T}\sum_{l=0}^{T}\frac{\mathrm{HD}(\mathrm{R}_i^{\mathrm{Ref}}(n),\mathrm{R}_i^{l}(n))}{n}$$

where $R_i^{Ref}(n)$ is the n-bit reference response at Day 0 from the ith PUF, k is the total number of PUF patterns and T is the number of trials and HD is the Hamming Distance (defined in the main text).
